# Case report and literature review: Orally ingested toothpick perforating the lower rectum

**DOI:** 10.3389/fsurg.2024.1368762

**Published:** 2024-02-16

**Authors:** Xingliang Zhang, Mei Xing, Shaoyang Lei, Wentao Li, Zijin Li, Yibing Xie, Chenyu Zhu, Shuqian Zhang

**Affiliations:** ^1^ Department of Graduate School, Hebei Medical University, Shijiazhuang, Hebei, China; ^2^ Department of Radiology, Hebei General Hospital, Shijiazhuang, Hebei, China; ^3^ Department of Radiology, First Teaching Hospital of Tianjin University of Traditional Chinese Medicine, Tianjin, China; ^4^ Department of Radiology, Yuebei People’s Hospital, Shaoguan, Guangdong, China; ^5^ Department of Graduate School, Hebei North University, Zhangjiakou, Hebei, China; ^6^ Department of Graduate School, North China University of Science and Technology, Hebei, China

**Keywords:** toothpick, foreign bodies, gastrointestinal perforation, rectum perforation, computed tomography

## Abstract

**Introduction:**

Most foreign bodies (FBs) can spontaneously pass through the gastrointestinal tract. Sharp FBs are believed to be able to puncture any part of the gastrointestinal tract, causing perforation and potentially secondary damage to adjacent organs.

**Case description:**

A 44-year-old man complained of having persistent dull pain in the perianal region. He was diagnosed with a toothpick impacted into the wall of the lower rectum after accepting a digital rectal examination of the lower rectum and a pelvic computed tomography (CT). The surgeon extracted the FB using vascular forceps guided by the operator’s index finger. The patient was discharged after intravenous ceftriaxone was given for 6 days. A follow-up pelvic CT performed 2 weeks after surgery revealed that the perirectal fat and muscles had already normalized.

**Conclusion:**

A systematic review of relevant literature from the past decade was performed to summarize the imaging features of an orally ingested toothpick perforating the gastrointestinal tract. The location of abdominal pain is an important clue for the diagnosis of toothpick perforation, and a CT examination is recommended as the first option for the detection of an ingested toothpick. Determining the location of the toothpick perforation and assessing the severity of local inflammation are important bases for the selection of treatment.

## Introduction

A variety of foreign bodies (FBs) are known to be ingested unintentionally through the mouth. Although the majority of FBs can spontaneously pass through the gastrointestinal tract without complications, some sharp FBs are believed to be able to puncture any part of the gastrointestinal tract, causing perforation and even secondary damage to adjacent organs (1, 2). Therefore, timely imaging examinations are necessary to identify complications caused by FBs in patients who are aware of having swallowed sharp and hard FBs (3). In this study, we describe a case of a 44-year-old man diagnosed with a toothpick impacted into the wall of the lower rectum and perform a systematic review of relevant literature from the past decade to summarize the imaging features of this disease entity.

## Case description

**Figure 1:**
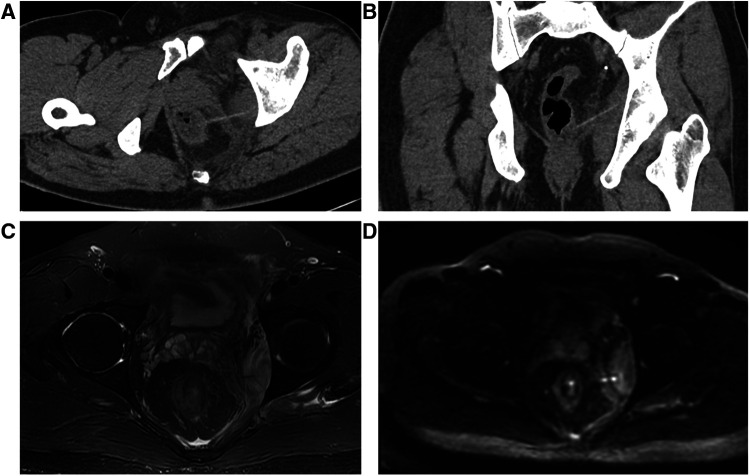
CT multiplanar reformation (MPR) (**A,B**) images showing that the toothpick in the distal rectal segment appears as a short linear hyperdense substance. One end is located in the left lateral wall of the rectum and penetrates the left levator ani muscle, while the other end penetrates the left internal obturator muscle. These two muscles are swollen with blurred surrounding fat. Axial T2WI (**C**) and DWI (**D**) of pelvic MRI reveals that there is a linear hypointensity in the left lateral wall of the lower rectum, consistent with the ingested toothpick, and the left levator ani and internal obturator muscles become thickened.

**Figure 2:**
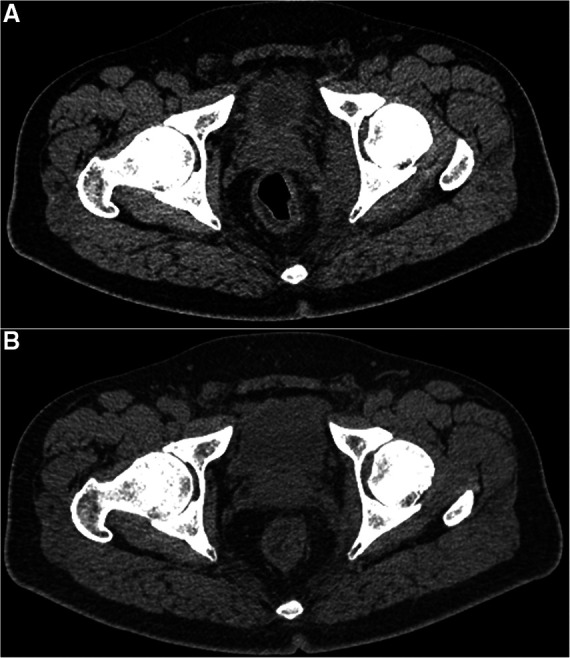
A comparison of axial pelvic CT images before (**A**) and 12 days (**B**) after removing the toothpick from the lower rectum indicates that the swelling of the two muscles on the left side of the rectum becomes thinned and the inflammatory infiltration in the surrounding fat space disappears.

A 44-year-old man presented to the gastrointestinal surgery clinic with a 1-week history of persistent dull pain in the perianal region, which worsened when standing and walking. Simultaneously, he developed a fever of 37.5°C. He was diagnosed with “diabetes mellitus type 2” one month previously and treated with pioglitazone hydrochloride and metformin hydrochloride tablets, following which he achieved good glycemic control. The abdomen was found to be soft with no tenderness when a physical examination was conducted in a supine position. An anal examination in the knee-chest position showed mild tenderness without redness and swelling around it. Upon a digital rectal examination at the level of the lower rectum, a sharp tip could be touched in the rectal wall at 9–12 o'clock with apparent tenderness. The latex glove was stained with blood and no purulent secretion was observed. A pelvic computed tomography (CT) demonstrated that the left wall of the lower segment of the rectum approximately 6 cm above the anal verge was thickened, and a linear hyperdensity of 46 mm [CT value: 108–134 Hounsfield units (HU)] was detected outside the rectal wall, which was considered a foreign body (Figures 1A,B). The FB was inserted into the left internal obturator muscle through the mesorectal fat and the left levator ani muscle. No bubble-like air shadow was detected around the FB or the adjacent fat space. The left levator ani muscle and the left internal obturator muscle were significantly thickened to approximately 7 and 19 mm, respectively, with poorly defined boundaries. The focal mesorectum and the fat space between the two muscles were blurred. A pelvic magnetic resonance imaging (MRI) showed that the left rectal muscle swelled and presented as increased signal intensity on the T2-weighted imaging (T2WI) and diffusion-weighted imaging (DWI), with a linear low signal penetrating the rectal wall to the left internal obturator muscle (Figures 1C,D). The patient stated that he got drunk ten days ago. On the day of the CT and MRI examination, the patient was placed in the supine lithotomy position and his anus was enlarged to four fingers under subarachnoid block anesthesia. A sharp FB was touched in the rectal wall at 2–3 o'clock approximately 6 cm above the anal verge. The surgeon held vascular forceps in his right hand, and guided by his left index finger, grasped and removed the FB along its long axis. No suturing was performed due to the fact that it was only a small wound with mild bleeding in the rectal mucosa. The FB was a wooden toothpick with a length of approximately 46 mm with two sharp tips. The patient was discharged after a single 2,000 mg intravenous dose of ceftriaxone per day was administered for 6 days. The patient made a return visit two weeks after the surgery, with no complaint of pain in the perianal region. A follow-up pelvic CT revealed that the left levator ani muscle and the left internal obturator muscle became thin with measurements of 5 and 10 mm, respectively, with clear boundaries and a gap in the local mesorectal fat (Figure 2).

## Literature review

**Figure 3:**
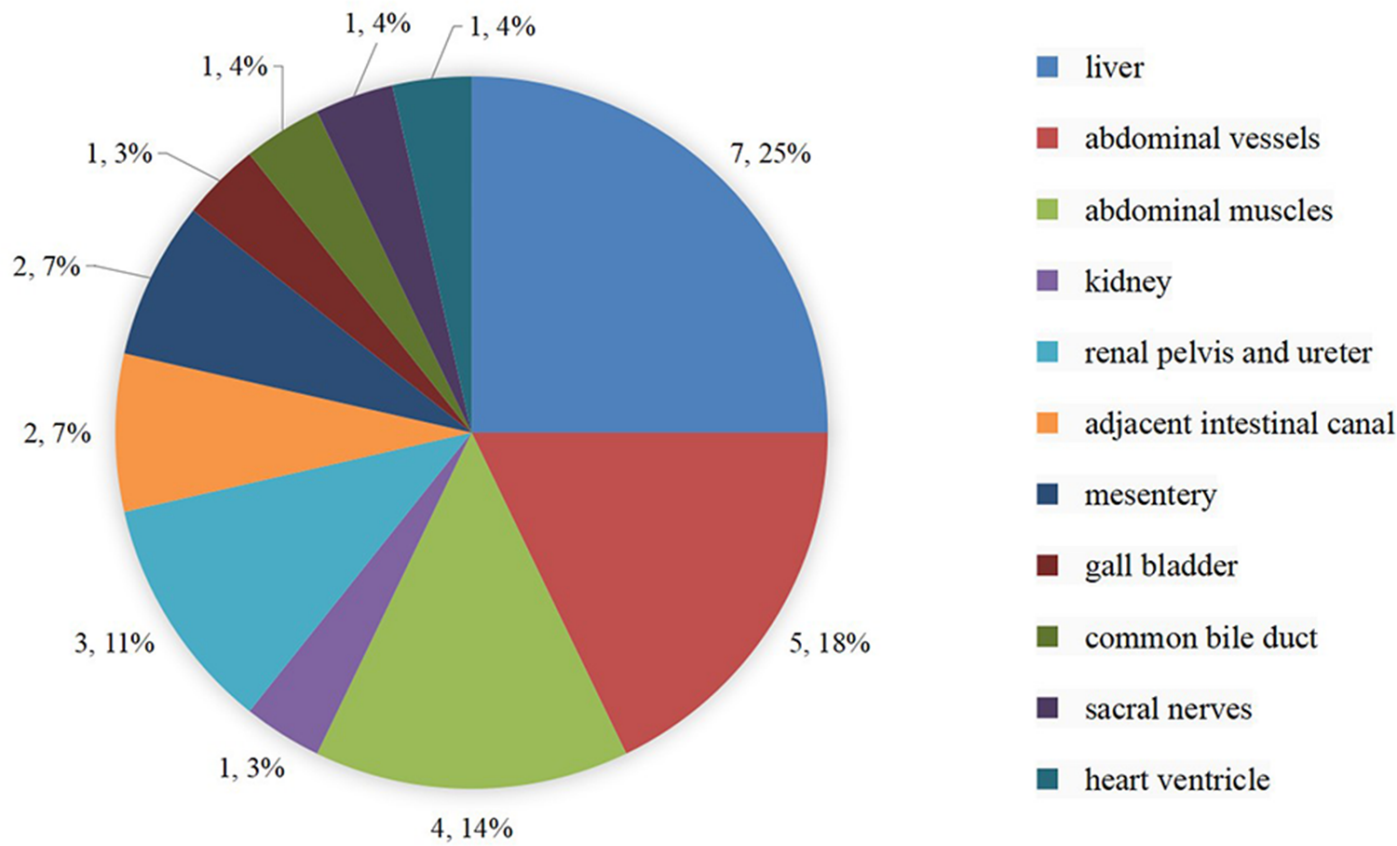
A summarization of secondary injuries to adjacent organs.

**Figure 4:**
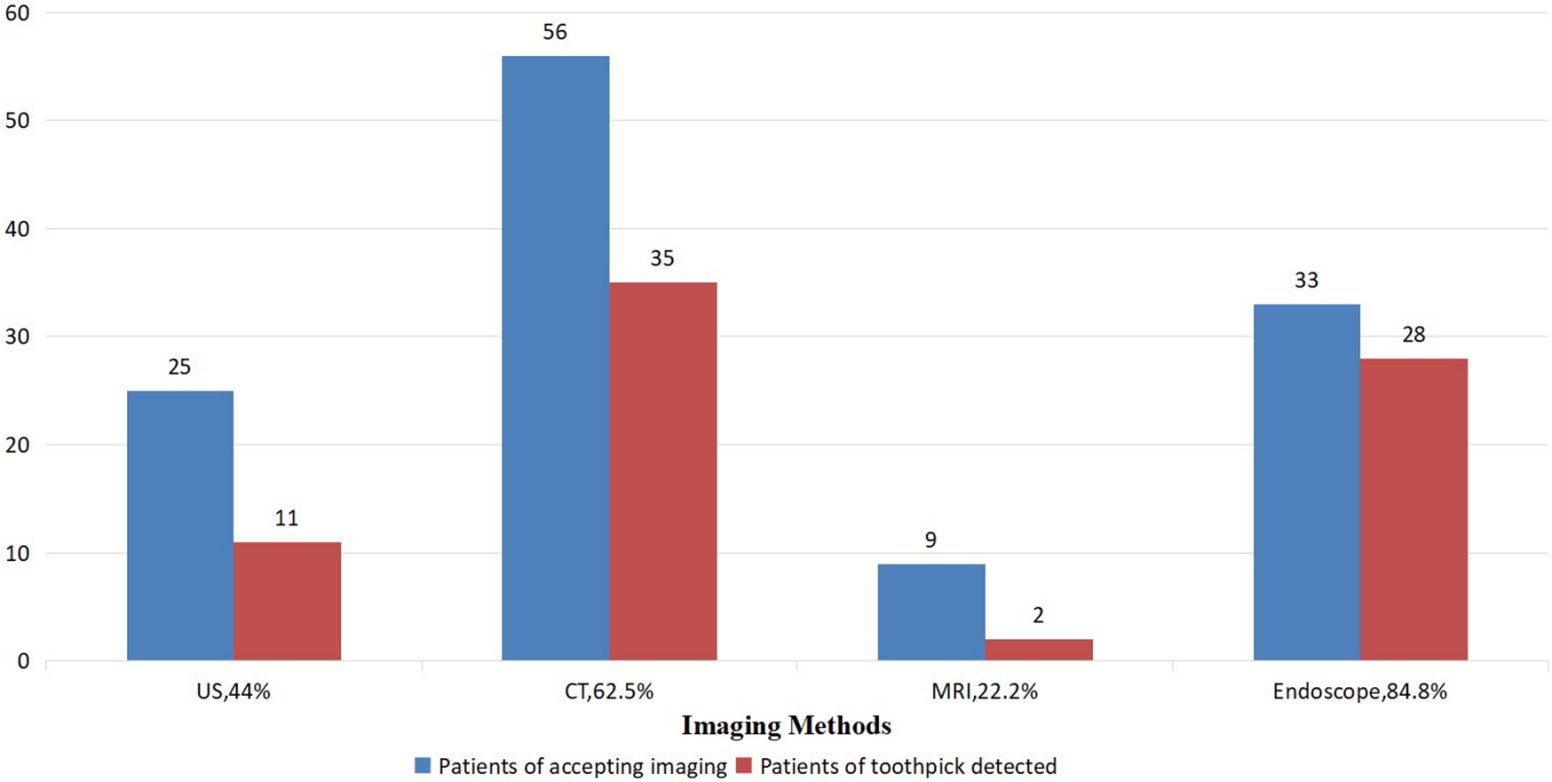
Sensibilities for different imaging methods before removing the toothpick.

A literature search was performed in the PubMed/MEDLINE database of case reports and original studies to identify potentially relevant articles that were published between 1 January 2013 and 31 August 2022. A different combination of search terms (Toothpick OR Perforation AND [Gastrointestinal Tract (MeSH term) or Alimentary Canal or Digestive Tract or Alimentary Tract]) was used to retrieve articles on gastrointestinal perforation without language restrictions, and these articles pertained to accidental ingestion of toothpicks in humans. A total of 59 case reports were yielded through the literature search, consisting of 68 patient cases and 70 toothpicks, since two patients had each ingested two toothpicks. No duplicate literature was included in this review, compared with a previous similar review (1) published online on 29 October 2013. Figures 3, 4 can be referred to for details. General information and summarization of toothpick perforation locations are available in the Supplementary Material.

Traditional x-ray radiography remains the primary imaging method for detecting ingested metallic foreign bodies. However, most patients are unaware of having swallowed an FB or are unable to recall the event. Only when symptoms such as abdominal pain, diarrhea and bloody stools, and high fever develop will they be admitted to the hospital. However, acute gastrointestinal bleeding or peritonitis secondary to the perforation of the digestive tract caused by some sharp FBs can be life-threatening. The interval between ingestion of FBs and admission to hospital is generally related to the distance that FBs travel through the digestive tract. The previous investigation reported a case of an FB remaining in the abdominal cavity for up to 6 months since the perforation of the digestive tract (2). Imaging methods play a crucial role in identifying FBs that perforate the digestive tract, especially those methods that provide cross-sectional images, such as CT and MRI.

Ingested non-metallic FBs have been found to be composed of a variety of materials, including bones (from poultry, domestic animals, fish, etc.), wood (matchsticks, toothpicks, chopsticks, etc.), and plastics (lighters, pen caps, straws, etc.). Thick bones can significantly attenuate x-rays and appear hyperdense on x-rays or CT. In contrast, since x-rays can penetrate plasticity, the diagnosis of plastic FB depends widely on the gas density within it. The detection of an ingested FB is facilitated by the density difference between the ingested FB and the surrounding contents of the gut lumen or abdominal organs. Toothpicks are one of the most common ingested FBs in the gastrointestinal tract in clinics due to their small shape and wide use in daily activities (3). Particularly for patients who are drunk or have an intellectual disability, or even for those who are bumped by others while picking teeth, toothpicks can easily enter the digestive tracts orally. After summarizing case reports from the past decade, we found that 59 reports mentioned gastrointestinal perforations, including 68 patient cases and 70 toothpicks in total. Perforation of the lower digestive tract occurred in 45 of 68 patients (66.2%). Endoscopy methods (including gastroscopy, endoscopic retrograde cholangio-pancreatography (ERCP), cystoscopy, and ureteroscopy) were performed to extract toothpicks in 27 patients (39.7%). The removal of ingested toothpicks was achieved by laparotomy in 40 patients (58.8%). One patient (1.5%) died of cervical cellulitis resulting from the perforation of a toothpick, and the toothpick was found near the pharyngo-esophageal junction at autopsy. It is, therefore, paramount that patients with ingested toothpicks undergo prompt and detailed tomographic imaging to prevent the occurrence of serious complications.

## Different imaging features of toothpicks

Ultrasound (US) is a common screening method for patients with abdominal pain due to its low cost and easy implementation. The toothpick presents as a sharp linear hyperechoic structure with posterior acoustic shadowing on the US (4). Regions of inflammation secondary to toothpick penetration appear as hypoechoic or hyperechoic masses with ill-defined boundaries, heterogeneous echoes, and abundant blood flow signals. Gas leak into the abdominal cavity caused by the penetration presents as a hyperechoic shadow and the fluid exudation around the thickened bowel wall presents as hypoechoic (5, 6).

Given that toothpicks are very thin and may be covered by surrounding inflammatory mass or fluid exudation (7), it is difficult for x-rays with low-density resolution to detect such small wooden FBs. As a result, x-ray is generally not considered an ideal tool for detecting ingested toothpicks, regardless of whether the patient is aware of it or not. Nevertheless, some indirect signs after perforation can be identified by x-rays, including free gas under the diaphragm and intestinal obstruction (8).

On CT images, the density of wooden FBs mainly depends on the species of the tree and wood surface coatings, and their CT values may range from −984 to −70 HU (9). Therefore, a wider window width (∼1,000 HU) and a lower center level (−500 HU) are recommended for optimal detection of wooden FBs (10, 11). Of note, the CT values of ingested wooden FBs may increase over time as the relatively dry wooden FBs will gradually absorb water from the surrounding digestive tract. In our patient case, the toothpick perforating through the lower part of the rectum presents as a thin, short, rod-shaped structure with a higher CT value (108–134 HU) than the surrounding soft tissue, indicating that the raw material for the toothpick has high density. This is consistent with the summarized results from the literature that most toothpicks present as high-density linear structures on CT images. Only one case reported that a toothpick (2 cm) penetrating the gastric posterior wall revealed a short linear gas density on the CT images (12), which may be attributed to the loose wood material of the toothpick. Moreover, 30.5% (18/59) of case reports providing photographs of removed toothpicks reveal that toothpicks retain their sharp tips and are not eroded by stomach acid or digestive fluids. This may account for the fact that most perforated toothpicks are made of relatively dense and hard wood and therefore have higher CT values than the surrounding soft tissue and tend to cause intestinal perforations. The regional inflammation in the intestinal wall caused by toothpick perforation can be identified on CT images as a thickened intestinal wall with fatty infiltration around the perforation region (7, 11). In addition, pneumoperitoneum is a significant indirect sign of perforation, which appears as focal extraluminal free gas bubbles adjacent to the perforated region (6, 13). Furthermore, toothpicks may also cause secondary damage to the neighboring organs after penetrating from the digestive tract. For instance, the perforated toothpick from the gastrointestinal tract can penetrate the liver parenchyma and cause liver abscesses, which present as a hypodense inflammatory infiltrate or as multilocular abscesses in the liver (14–16). A contrast-enhanced CT is recommended for the diagnosis of liver abscesses. There are two mechanisms of liver abscesses caused by ingested toothpicks (17). One is caused by direct insertion into the liver after perforating from the upper gastrointestinal tract; the other is caused by a hematogenous dissemination of inflammatory substances. In addition, small bowel obstruction is also a common complication of ingested toothpicks. The linear hyperdensity, consistent with a toothpick appearance, is often detected at the proximal stenotic segment of the obstructed small bowel, which appears as multiple air–fluid planes in the lumen of the proximal small intestine accompanied by mesenteric fat turbidity on CT images (8, 18). Moreover, secondary injuries after toothpick perforation may also involve adjacent blood vessels, muscles, the biliary tract, and urethra, leading to various complications such as thrombosis and hematuria (19, 20).

Compared with CT, MRI is not commonly recommended as a routine method to detect toothpick perforation. An accidental ingestion of toothpicks can occasionally be detected by MRI. The toothpicks show a linear hypointensity on both T1-weighted imaging (T1WI) and T2WI (21). When toothpick perforation results in local inflammation or abscess, the linear hypointensity can be easily distinguished from the surrounding high-signal abscess on T2WI.

## Diagnostic assessment

The key point of the diagnosis in the case of our patient in this study was the detection of a linear hyperdensity with a length of 46 mm outside the rectal wall by CT, which provided the morphological clue to speculate about an ingested toothpick.

## Discussion

Previous literature suggested that ingested toothpicks lead to digestive tract perforation with a 79% probability rate (1), mostly in the colon and rectum. The size, sharpness, and shape of the ingested foreign body are some characteristics that should be taken into consideration when a medical professional has to deal with this condition. The risk of injury increases when the size of the object is more than 5 cm or the object has a pointed shape (22, 23). Approximately 47.1% (33/70) of colorectal perforations were caused by toothpicks in the last decade, indicating that toothpicks could pass through the narrow ileocecal valve or possibly be discharged through the anus. Only approximately 8.6% (6/70) of ingested toothpicks could reach the end of the digestive tract. In our patient, the toothpick perforated from the lower rectum approximately 6 cm above the anal verge, which was rare in the reported literature. The patient developed symptoms 3 days after ingesting the toothpick, which is consistent with the time range of excretion from the anus since oral ingestion (24). Toothpick perforation could involve almost the entire digestive tract according to the literature review. Although 82% (56/68) of patients present with abdominal pain as the initial symptom, the location of the abdominal pain is highly consistent with the site of the gastrointestinal perforation. Therefore, it is recommended to perform detailed imaging examinations based on the areas where patients’ abdominal pains originate. Given that the ingested toothpick can remain in the abdomen for up to 6 months (2), radiologists should pay more attention to the indirect signs of FB perforation, including free gas, thickened intestinal wall, blurred fat space, abscess formation, and intestinal obstruction.

Because of the low attenuation of x-ray and the interference of intestinal inflammation, only 5.5%–15.0% of wooden FBs could be identified by x-ray (25), and the sensitivity rates of US and CT in detecting toothpicks were 22.0% and 42.6%, respectively (1). The detection rates for the toothpicks summarized in this review were 44.0% by ultrasound and 62.5% by CT, respectively, both of which were higher than those in the earlier literature reviews, suggesting that the improved image resolution of the examinations contributes to the detection of FBs. In addition, the review suggested that MRI had a detection rate of 22.2% for toothpicks, which was similar to US, but lower than x-ray, and therefore was not recommended as the first option. Although toothpicks showed a linear high echo with posterior acoustic shadows on US (5), the detection rate of toothpicks could be reduced by the interference of the intestinal gas. Based on a proper adjustment of window width and window level (10) and a reasonable application of three-dimensional reconstruction technology (15), CT can clearly display toothpicks, inflammation, abscess, and other secondary changes in surrounding tissues, and is regarded as the first choice for the clinical diagnosis of FBs in the digestive tract.

Recent literature over the past decade has shown that toothpick perforations caused up to 40.0% of secondary injuries, which were common in the liver, abdominal blood vessels, abdominal muscles, kidneys, ureters, intestines, and mesentery. In the treatment of toothpick perforations, sufficient attention should be paid to secondary injury to the adjacent organs. Treatment under endoscopy is the preferred method for those patients with non-migrated toothpicks in the proximal and distal segments of the gastrointestinal tract, due to its mini-invasive feature (26, 27). The laparoscopic method before open surgery can be performed safely for removing an ingested FB. A laparoscopic minimally invasive surgery should be preferred to open surgery due to its advantages (28). Surgery is commonly done in the small intestine and for retrieving migrated toothpicks owing to sufficient exposure of the surgical area, ease of detection of FBs and convenient extracting operations. In the course of the operation through endoscopy, the complete removal of the toothpick should be confirmed. If the toothpick cannot be completely removed, or if there is a residue due to a broken toothpick, surgical laparotomy is the only option. The surgeon cannot use endoscopy if the toothpick is located outside the wall of the lower rectum and cannot be seen through the lumen. In these cases, the toothpick can be removed by using vascular forceps along with palpation because of the short distance between the lower rectum and the anus, an operation that cannot be achieved under endoscopy.

## Conclusion

The location of abdominal pain is an important clue for the diagnosis of toothpick perforation, and a CT examination is recommended as the first option for the detection of ingested toothpicks. The toothpick commonly appears as a linear hyperdense substance on CT images, while indirect signatures, including extraintestinal air bubbles and inflammatory fat infiltration, provide a significant advantage for diagnosis. Determining the location of the toothpick perforation and assessing the severity of local inflammation are important for the selection of treatment.

## Scope statement

The oral toothpick ingested by the patient in this case could not be completely discharged through the anus but instead perforated and displaced into the left pararectal space. Such a situation occurs in less than one-tenth of reported cases. The location of the abdominal pain generated usually indicates the presence of perforation, which has been confirmed by previous literature. A careful reading of radiological images based on the location of abdominal pain is essential for the detection of the toothpick. The appearance of free gas, a thickened intestinal wall, blurred fat space, abscess formation, and even intestinal obstruction may help indicate perforation. Due to the window adjustment technique and three-dimensional reconstruction using volume data, CT has prominent advantages in terms of displaying the direct and indirect signs mentioned above, as well as the secondary damage to the surrounding abdominal organs. Laparoscopic surgery is the preferred minimally invasive method for the treatment of complete toothpick perforation, while partial toothpick perforation can be removed by endoscopy. Imaging evaluation could provide sufficient evidence for selecting reasonable treatment methods.

## Data Availability

The original contributions presented in the study are included in the article/Supplementary Material, and further inquiries can be directed to the corresponding author.
