# Supplementary material for: Case report and literature review: Orally ingested toothpick perforating the lower rectum
Source: Front Surg. 2024 Feb 16;11:1368762. doi: 10.3389/fsurg.2024.1368762 (PMC10904550; doi:10.3389/fsurg.2024.1368762)
Supplement: Supplementary file 1 [file Table1.docx]

**Table S1 General Information**

| **Literature type** | **Total (n=59)** |
| --- | --- |
| case, n (%) | 55 (93.2) |
| case series, n (%) | 4 (6.8) |
|  |  |
| **Patients** | **Total (n=68)** |
| Age, average (range) | 51 (9-90) |
| Male, n (%) | 50 (73.5) |
|  |  |
| **History of ingesting toothpick** | **Total (n=68)** |
| cannot recall, n (%) | 41 (60.3) |
| can recall, but cannot confirm toothpick ingested, n (%) | 18 (26.5) |
| can confirm toothpick ingested, n (%) | 9 (13.2) |
|  |  |
| **special condition** |  |
| in a state of intoxication, n (%) | 7 (10.3) |
| drug takers, n (%) | 1 (1.5) |
| with habit of picking teeth with toothpick, n (%) | 6 (8.8) |
| with history of taking food or drink with toothpick, n (%) | 10 (14.7) |
|  |  |
| **Initial Symptom** |  |
| abdominal pain, n (%) | 56 (82.4) |
| fever, n (%) | 15 (22.1) |
| nausea, n (%) | 8 (11.8) |
| diarrhea, n (%) | 4 (5.9) |
| constipation, n (%) | 4 (5.9) |
| perianal pain, n (%) | 3 (4.4) |

**Table S2 Summarization of Toothpick Perforation Sites**

|  | **Total (n=70)** |
| --- | --- |
| **Upper Digestive Tract** | **23 (32.9)** |
| pharyngo-esophageal junction, n (%) | 1 (1.4) |
| stomach, n (%) | 11 (15.7) |
| duodenum, n (%) | 11 (15.7) |
|  |  |
| **Lower Digestive Tract** | **47 (67.1)** |
| **small intestine** | **14 (20.0)** |
| without confirming in jejunum or ileum, n (%) | 7 (10.0) |
| ileum, n (%) | 5 (7.1) |
| ileocecal junction, n (%) | 2 (2.9) |
| **colorectum** | **33 (47.1)** |
| colon (without confirming any segment）, n (%) | 3 (4.3) |
| cecum, n (%) | 4 (5.7) |
| appendix, n (%) | 2 (2.9) |
| hepatic flexure of colon, n (%) | 2 (2.9) |
| splenic flexure of colon, n (%) | 1 (1.4) |
| left hemicolon, n (%) | 1 (1.4) |
| sigmoid, n (%) | 14 (20.0) |
| rectum, n (%) | 6 (8.6) |
